# Ivermectin: A Multifaceted Drug With a Potential Beyond Anti-parasitic Therapy

**DOI:** 10.7759/cureus.56025

**Published:** 2024-03-12

**Authors:** Baneet Kaur, Cyril Blavo, Mayur S Parmar

**Affiliations:** 1 Department of Medicine, Dr. Kiran C. Patel College of Osteopathic Medicine, Nova Southeastern University, Clearwater, USA; 2 Department of Public Health, Dr. Kiran C. Patel College of Osteopathic Medicine, Nova Southeastern University, Clearwater, USA; 3 Department of Foundational Sciences, Dr. Kiran C. Patel College of Osteopathic Medicine, Nova Southeastern University, Clearwater, USA

**Keywords:** nuclear factor kappa-light-chain enhancer of activated b cells (nf-κb), malaria, anti-cancer, anti-viral, anti-inflammatory, anti-parasitic, ivermectin

## Abstract

Ivermectin was first discovered in the 1970s by Japanese microbiologist Satoshi Omura and Irish parasitologist William C. Campbell. Ivermectin has become a versatile pharmaceutical over the past 50 years. Ivermectin is a derivative of avermectin originally used to treat parasitic infections. Emerging literature has suggested that its role goes beyond this and may help treat inflammatory conditions, viral infections, and cancers. Ivermectin’s anti-parasitic, anti-inflammatory, anti-viral, and anticancer effects were explored. Its traditional mechanism of action in parasitic diseases, such as scabies and malaria, rests on its ability to interfere with the glutamate-gated chloride channels in invertebrates and the lack of P-glycoprotein in many parasites. More recently, it has been discovered that the ability of ivermectin to block the nuclear factor kappa-light-chain enhancer of the activated B (NF-κB) pathway that modulates the expression and production of proinflammatory cytokines is implicated in its role as an anti-inflammatory agent to treat rosacea. Ivermectin has also been evaluated for treating infections caused by viruses, such as SARS-CoV-2 and adenoviruses, through inhibition of viral protein transportation and acting on the importin α/β1 interface. It has also been suggested that ivermectin can inhibit the proliferation of tumorigenic cells through various pathways that lead to the management of certain cancers. The review aimed to evaluate its multifaceted effects and potential clinical applications beyond its traditional use as an anthelmintic agent.

## Introduction and background

Since its discovery in the 1970s by the Japanese microbiologist Satoshi Omura and Irish parasitologist William C. Campbell, ivermectin has become a versatile pharmaceutical. While well recognized for its use in diverse clinical settings, it has also had a profound humanitarian impact, treating millions of people, particularly the most impoverished worldwide [1]. Ivermectin is a derivative of avermectin and is a macrocyclic lactone compound. The avermectins are isolated from the soil bacterium Streptomyces avermitilis [2]. Since its inception, ivermectin has revolutionized the treatment and control of several parasitic infections.

Ivermectin was made commercially available by the U.S.-based pharmaceutical company Merck Sharp and Dohme (MSD) for use in veterinary medicine in 1981 [1]. Following its successful application as a powerful anthelmintic agent in animals, ivermectin was recognized by scientists for its potential against a similar human pathogen, Onchocerca volvulus. The drug was then developed and tested for its antiparasitic effects in humans in a series of clinical trials [3-8]. These efforts culminated in the approval of ivermectin for human use, marking its introduction for therapeutic use in humans in the late 1980s [1,3]. Ivermectin was first used to treat onchocerciasis, commonly known as river blindness, and has evolved as an agent of choice for a broad range of parasitic infections.

The mode of action of ivermectin is based on its ability to selectively inhibit glutamate-gated chloride channels (GluCls) at nanomolar concentrations in invertebrates [9-13]. These channels are not expressed in vertebrates [2]. Inhibition of these channels affects feeding, motility, and reproduction [2]. At higher concentrations, ivermectin can interact with several receptors in both invertebrates and vertebrates, including gamma-aminobutyric acid, glycine, histamine, and nicotinic acetylcholine receptors [2]. Through its effects at high and low concentrations, ivermectin induces the death of parasitic agents. In humans and other vertebrates, the P-glycoprotein (P-gp), also known as the multidrug resistance protein 1 (MDR1), exerts a protective effect [14,15]. It is expressed in the blood-brain barrier and acts as an efflux pump, shunting ivermectin out of the central nervous system (CNS) [15]. In some animals, such as dogs and horses, the absence of P-gp can lead to toxicity, with manifestations of drowsiness, coma, and even death [2].

Oral ivermectin is the only approved route of administration for human use; however, recently, it has been demonstrated to be efficacious topically [16-18]. It has a well-established safety profile in adults with a significantly low rate of adverse reactions [19]. An exception to this is its well-documented risk in treating loiasis and onchocerciasis, where the death of high loads of microfilaria may lead to severe encephalopathy [19]. Despite these concerns, there is literature documenting its safety in young children [2]. However, there is still a lack of support in the literature regarding its safety in pregnant women, so its use is ill-advised in this population.

Today, ivermectin is used to treat a wide range of nematode infections in humans, including onchocerciasis, strongyloidiasis, loiasis, ascariasis, filariasis, cutaneous larva migrans, gnathostomiasis and trichuriasis, and arthropod infestations caused by Sarcoptes scabiei, Demodex, and Pediculosis [1]. Ivermectin is currently being evaluated and under extensive study in clinical trials for its potential use in treating mosquito-borne parasitic infections and as a complementary strategy for reduction through mass drug administration [20-24]. Although its antiparasitic effects are well documented, the anti-inflammatory activity of ivermectin has recently been uncovered [25]. This follows its approval by the U.S. Food and Drug Administration (FDA) and the European Union for the treatment of rosacea. Emerging literature highlights the potential use of ivermectin as a promising candidate for anticancer therapy [26-29]. Several studies have addressed its antiviral activity against a diverse range of viruses, leading to its exploration for use in treating SARS-CoV-2 during the most recent global pandemic [30].

The field of medicine is constantly evolving, and it is important to continue updating our understanding of drug pharmacology and its potential applications. Ivermectin, which is traditionally used as an anthelmintic agent, has been found to have a range of effects beyond its initial antiparasitic use. This review article aimed to evaluate the multifaceted effects of ivermectin treatment and explore its potential clinical application for other diseases. Given the increasing evidence of the broad-spectrum activity of ivermectin, it is timely and necessary to carry out this comprehensive review, potentially leading to more effective and versatile therapeutic strategies in the future.

## Review

In Table 1, we summarize the authorized and recommended uses of ivermectin by the FDA and World Health Organization (WHO), as well as its investigational status for various conditions.

**Table 1:** Ivermectin, a multifaceted drug. Summarizes the FDA-approved and investigational status of ivermectin for various conditions. Please note that this information is for informational purposes only and does not constitute medical advice. Always consult with a healthcare professional for any questions or concerns. Also, research is ongoing, so it’s important to stay informed about the latest developments. FDA, U.S. Food and Drug Administration; WHO, World Health Organization

Therapeutic indications	FDA-approved	Investigational research	Keynotes
Parasitic infections			
Intestinal strongyloidiasis (caused by Strongyloides stercoralis)	✔️		
Onchocerciasis (river blindness caused by Onchocerca volvulus)	✔️		Only active against the tissue microfilariae of Onchocerca volvulus. No activity against adult O. volvulus.
Lymphatic filariasis (caused by Wuchereria bancrofti)	X	✔️	Important medication (WHO recommendation) as a part of mass drug administration regimen. Ivermectin is preferred to treat Lymphatic filariasis in areas with onchocerciasis. Primarily in combination with other drugs, effective against the circulating microfilariae of W. bancrofti.
Scabies (caused by Sarcoptes scabiei)	X	✔️	Medication for scabies is now on the WHO essential medicines list. Ivermectin is not FDA-approved for scabies but is effective in treating infections caused by S. scabiei (scabies), crusted (Norwegian) scabies, or superinfected scabies. Ivermectin can be administered orally or topically using a 0.5% lotion for these conditions.
Skin conditions			
Rosacea (inflammatory skin condition)	✔️		Topical application (product: 1% cream). Effective for papules and pustules (strong clinical trial evidence, but only somewhat effective for flushing (weak clinical trial evidence).
Other potential uses (under investigation)			
COVID-19 (caused by SARS-CoV-2)	X	✔️	Large-scale studies show inconclusive or negative results.
Adenoviruses	X	✔️	Early-stage research
Autoimmune diseases	X	✔️	Limited and preliminary research
Cancer	X	✔️	Early-stage research

Antiparasitic activity

The introduction of ivermectin as an antiparasitic agent in veterinary medicine paved the way for its use in the treatment of parasitic infections in humans, which was its first established and known use in humans. Ivermectin was initially introduced to treat onchocerciasis and has been shown to successfully control and eliminate infection through mass drug administration [2]. It has indications for use across a wide range of other parasitic infections including, but not limited to, strongyloidiasis, loiasis, ascariasis, filariasis, cutaneous larva migrans, gnathostomiasis, scabies, and pediculosis [1]. Approximately 250 million people use ivermectin annually to control various parasitic diseases [16]. The mechanism of action of ivermectin in parasites relies on its ability to interfere with glutamate-gated chloride channels in invertebrates and the lack of P-gp in many parasites [9-13]. The next section will focus on the use of ivermectin in the treatment of scabies, where it has been evaluated as a first-line agent, and examine its potential as an antimalarial agent.

Scabies

Scabies is a parasitic skin infestation caused by the mite Sarcoptes scabiei. It has recently been noted that its incidence is increasing in humans [31-33]. Pregnant female mites dig deep into the upper layer of human skin, the stratum corneum, and reside there for a lifetime of four to six weeks [34,35]. During this period, they lay eggs that hatch in two to three days, producing larvae that mature within 9 to 17 days [34,35]. Male mites die shortly after, while the presence of female mites causes symptoms. Clinical signs and symptoms generally worsen at night, including a significantly pruritic rash that is papular or papulo-esicular and often symmetrically distributed [35]. Commonly affected areas include the axillae, interdigital spaces of the hands and feet, waistlines, and buttocks. Microscopic scabies mite is transmitted primarily through direct and prolonged skin-to-skin contact with an infected person (Centers for Disease Control and Prevention). An infected host can spread scabies even in the absence of symptoms. It is crucial to note that the infestation is a strictly human-to-human transmission and that animals do not serve as vectors for human scabies.

First-line therapy for scabies has historically been topical permethrin, a synthetic pyrethrin, although recent reports indicate the decreasing efficacy of this first-line agent [36,37]. Permethrin works by disrupting the sodium channel current across the neuronal membranes of mites. This disruption leads to delayed repolarization, resulting in paralysis and, eventually, death of the mites. Other treatment options for scabies include topical crotamiton, benzyl benzoate, 1% lindane lotion, or 10% sulfur [37]. Oral and topical ivermectin are approved treatment options for scabies [35,38]. The role of ivermectin against scabies is based on its mode of action as an antiparasitic agent that selectively inhibits glutamate-gated chloride channels (GluCls) at lower concentrations in parasites. Through the inhibition of these channels, functions such as feeding, motility, and reproduction are affected by the parasites and can lead to death.

A 2018 systematic review and meta-analysis conducted by Dhana et al. found that oral ivermectin was less effective than 5% topical permethrin [39]. The authors reported that the combination therapy comprising oral ivermectin and topical permethrin is safe and can even enhance the efficacy of each agent. The authors also noted that topical ivermectin may have a similar efficacy profile to topical permethrin, but additional conclusive studies still need to be conducted. The use of oral ivermectin needs to be further explored, particularly in resistance treatment to topical permethrin or compliance issues. Oral ivermectin, topical ivermectin, and 5% permethrin were well tolerated and associated with low treatment failure rates [39]. Oral ivermectin was compared to topical 1% lindane lotion, an organochloride insecticide, in a 2012 study conducted by Mohebbipour et al. At the one-week interval, it was found that a single dose of oral ivermectin was as effective as twice the application of 1% lindane lotion [40]. However, two doses of ivermectin were shown to be superior to 1% lindane lotion at the four-week follow-up. These studies demonstrate the potential of oral and topical ivermectin for effective scabies treatment. Notably, since ivermectin is the only oral agent currently available for the treatment of scabies, where there are cost issues, lack of desire to use topical agents, poor compliance with topical agents, or resistance to first-line therapies such as permethrin, ivermectin may be a reasonable choice for the treatment of scabies.

Malaria

There has been growing interest in the potential use of ivermectin for malaria control. Malaria is a fatal parasitic disease transmitted by the bite of an infected female Anopheles mosquito [41]. Of the five species of Plasmodium responsible for malaria, Plasmodium falciparum is considered the most lethal/predominant [41]. Patients often report symptoms such as fever, chills, headache, sweats, and malaise and may show signs of jaundice. Severe malaria can result in organ failure. Treatment commonly involves antimalarial drugs, such as chloroquine or artemisinin-based combination therapies (ACTs) [41].

The potential role of ivermectin in the management of malaria is through vector control and transmission. The key to controlling infection is to prevent human-to-vector transmission of the Plasmodium parasite. The Anopheles mosquito relies on glutamate-gated chloride channels for sensory and motor function [2]. Since ivermectin can selectively inhibit these glutamate-gated chloride channels, these essential functions are inhibited in Anopheles mosquitoes, leading to death [42]. In humans, oral ivermectin has been shown to achieve serum concentrations that can kill mosquitoes after blood meal, preventing transmission of the plasmodium parasite to another person [43]. The promising effect of ivermectin may be augmented via its sporontocidal effect [43]. Current modeling and field studies conducted by Bellinger et al. suggest that co-administration of ivermectin and artemisinin combination therapies (ACTs) may be effective by targeting mosquitocidal effects and malaria transmission [43]. The Repeat Ivermectin Mass Drug Administration for Control of Malaria (RIMDAMAL) study evaluated the impact of ivermectin on the cumulative incidence of uncomplicated malaria [44]. The results showed a reduction in the incidence of uncomplicated malaria in children <5 years of age [44]. However, the statistical methods used for these analyses have been questioned [45,46]. More extensive trials are now in place and ongoing to retrieve more conclusive data on the role of ivermectin in malaria control [47].

Anti-inflammatory activity

The role of ivermectin as an anti-inflammatory agent has only recently been understood. This is an integral part of its use as an antihelminthic agent. Ivermectin is now known to play an immunomodulatory role that suppresses inflammatory responses in humans [48]. It inhibits liposaccharide (LPS)-induced cytokine production [49]. Toll-like receptors on macrophages recognize LPS, which leads to sequential expression and secretion of proinflammatory cytokines such as tumor necrosis factor α (TNF-α), interleukin 6 (IL-6), and integrin-β6 [48]. This is made possible by the ability of ivermectin to block the nuclear factor kappa-light-chain enhancer of the activated B (NF-κB) pathway that modulates the expression and production of proinflammatory cytokines [49]. This prevents toll-like receptor 4 from initiating a cascade that leads to the production of proinflammatory cytokines. It is suggested that this mode of action explains why ivermectin may be helpful in intensive care unit settings with increased risks of LPS-mediated bacterial infections [50]. Ali et al. demonstrated that increased levels of IL-17 in rosacea led to the production of proinflammatory cytokines such as IL-1 and TNFα through the nuclear factor kappa-light-chain enhancer of activated B (NF-κB) pathway, which is again inhibited by ivermectin [51]. This prevents NF-kB from allowing IL-17 to produce proinflammatory cytokines such as IL-1 and TNFα. In addition, ivermectin has been shown to inhibit signal transducer and activator of transcription 3 (STAT-3), which is responsible for upregulating proinflammatory markers in macrophages [50,52]. The ability of ivermectin to modulate the production of proinflammatory cytokines plays a vital role in a wide range of pathological conditions.

Rosacea

Rosacea is a chronic, progressive, inflammatory skin condition. With a prevalence of almost 5.5%, it is a disorder that can cause a great disease burden, including its impact on self-esteem and the quality of life of affected individuals [53]. Rosacea usually involves the face and initially presents with recurrent erythema, telangiectasia, and flushing [53]. As the disease progresses, it presents with persistent erythema with papulopustules, follicular papules, and pustules [54]. Ultraviolet radiation, spicy foods, stress, and alcohol are well-known triggers for this disease [51]. The pathogenesis of rosacea, although not fully understood, is considered multifaceted. Among the significant mechanisms postulated are abnormal immune response and neurovascular dysregulation [51]. In 2019, Ali et al. discussed the likelihood of a pivotal role of IL-17 in the pathogenesis of rosacea [51]. This emerging theory is already a target of several current therapeutic agents used to treat rosacea.

Currently, there is no cure for rosacea. Most approved agents on the market provide symptomatic relief. Topical metronidazole, azelaic acid, and ivermectin are all approved for rosacea treatment [53]. Low-dose doxycycline and isotretinoin also contribute to the management of rosacea [54]. A pulsed dye laser (PDL) has been used successfully to treat the vascular components of rosacea [55]. Ivermectin plays a role in the treatment of rosacea through its ability to suppress inflammatory markers and responses. By inhibiting the nuclear factor kappa-light-chain enhancer of activated B cells (NF-κB) pathway, ivermectin can prevent the cascade of IL-17 and reduce the expression and secretion of proinflammatory cytokines such as IL-1 and TNFα [51]. Patients with rosacea have been found to have increased levels of Demodex mites on their skin [56]. The role of ivermectin as an antiparasitic agent is thought to play an additional role in the treatment of rosacea by reducing Demodex mite infestation and providing symptomatic relief [56].

A 2018 systematic review by Ebbelaar et al. found that topical ivermectin was an effective option for the treatment of papulopustular rosacea. It appeared to be more effective than topical metronidazole [18]. However, approximately two-thirds of the patient population relapsed within 36 weeks after discontinuing either treatment. Another study by Osman et al. sought to determine the effectiveness of PDL alone versus in combination with 1% topical ivermectin in the treatment of rosacea. At the three months of follow-up, patients who received the combination treatment had a better clinical improvement than those with PDL therapy alone [55]. However, the difference was not statistically significant. The authors concluded that the PDL may be more effective when combined with topical ivermectin 1% [55]. These studies demonstrate the potential of ivermectin as a combination therapy for rosacea and the need to explore the use of ivermectin for the treatment of relapsing rosacea.

Antiviral activity

Over the past decade, much research has investigated the potential of ivermectin as an antiviral agent. Viruses are known to have the ability to transport themselves into the nucleus and take over host activity. This ability is often made possible by nuclear imports of viral proteins, such as importin (IMP)-α/β1 hetero-dimer [57]. Ivermectin is one of those agents that has been found to target the IMP-α/β1 interface by inhibiting the binding of IM-α/β1 to several viral proteins [57]. Through this mechanism, ivermectin may have antiviral effects on viruses that rely on this interface, such as human immunodeficiency virus-1 (HIV-1), dengue (DENV), Zika (ZIKV), West Nile virus (WNV), Venezuelan equine encephalitis virus, chikungunya, and SARS-CoV-2 (COVID-19) [58]. Furthermore, ivermectin has been shown in some studies to be an inhibitor of viral replication [59-62]. The below section will focus on the contentious use of ivermectin in the management of COVID-19 and its application in the treatment of human adenoviruses.

SARS-CoV-2

In the context of the most recent pandemic, ivermectin garnered significant attention due to its potential therapeutic implications in the management of COVID-19. Its mode of action in the treatment of COVID-19 is based on the fact that ivermectin can inhibit viral protein transportation through IMP-α/β1 [57]. In addition, the anti-inflammatory characteristic of ivermectin may explain its potential impact on infectious agents such as COVID-19. A cytokine storm in severe COVID-19 has been described, which involves STAT-3 upregulation of proinflammatory cytokines [50]. Ivermectin has been shown to inhibit STAT-3, so it may help treat severe cases of COVID-19. In vitro studies of ivermectin have demonstrated that it kills SARS-CoV-2 in 48 hours [30]. The inhibition of SARS-COV-2 by ivermectin in vitro was evaluated by quantitation of viral load using real-time PCR [30]. Marques et al. reported that there are currently 81 clinical trials being carried out worldwide regarding the clinical use of ivermectin [30]. Marques et al. cite a single isolated study in which patients diagnosed with SARS-CoV-2 who received at least one dose of ivermectin during hospitalization were associated with lower mortality, particularly among those with increased oxygen and ventilatory requirements [30]. Jans et al. cite a study in Bangladesh that found that none of the 115 patients who received a single dose of ivermectin developed cardiovascular or pulmonary complications, while in 133 controls, 9.8% developed pneumonia, and 1.5% had an ischemic stroke [58]. The same study revealed that patients treated with ivermectin transitioned to a COVID-19-negative status in a short time, with a median of four days compared to 15 days in controls.

However, Deng et al. conducted a meta-analysis of randomized controlled trials. They found that the use of ivermectin was not associated with a reduction in viral clearance time, hospitalization duration, mortality incidence, or mechanical ventilation [63]. These authors cite the low to moderate quality of the evidence in this search and identify the need for further trials to clarify the use of ivermectin in the treatment of COVID-19. In a recent meta-analysis, Marcolino et al. evaluated 25 randomized clinical trials totaling over 6,000 patients and found that ivermectin does not reduce mortality risk or mechanical ventilation requirements [64]. Furthermore, a 2023 meta-analysis on the use of ivermectin for post-exposure prophylactic measures in COVID-19 management revealed that ivermectin did not exert a protective effect on this population [65]. This study by Hu et al. has observed a plausible protective effect of ivermectin in preexposed populations. However, the authors have cautioned that the results of this study should be interpreted with caution due to the poor quality of evidence in this subset of patients [65]. This suggests that further research is warranted to verify these findings and determine the potential utility of ivermectin in this context. Although isolated randomized clinical trials may show some benefit in some studies, the most recent large-scale and meta-analyses studies suggest that the role of ivermectin in the treatment of COVID-19 in humans is ineffective and inconclusive. The FDA has not authorized or approved the use of ivermectin for preventing or treating COVID-19 in humans or animals.

Adenoviruses

Human adenoviruses are generally known to cause mild symptoms, but individuals who are immunocompromised or vulnerable (such as the pediatric population) can develop severe disseminated disease [59]. One of the challenges is that there is currently no known effective antiviral agent to treat diseases caused by adenovirus. Adenoviruses are also known to rely on the importin α/β1 interface to gain access to the nuclear envelope [59]. This is one method by which ivermectin may work with adenovirus. King et al. also found that ivermectin inhibits human adenovirus C5 (HAdV-C5) early gene transcription, genome replication, early and late protein expression, and even the production of infectious viral progeny [59]. The ivermectin inhibition of the overall production of infectious HAdV-C5 progeny was dose-dependent [59]. Ivermectin also inhibited genomic replication of human adenovirus B3 (HAdV-B3), which has been linked to several recent outbreaks. However, it does not affect the human adenovirus E4 (HAdV-E4) [59]. King et al. have also reported findings of ivermectin affecting the binding of viral E1A proteins to IMP-α without affecting the IMP-α/β1 interaction [59]. It is important to note that these findings have been demonstrated in vitro. The potential of ivermectin in the management of human adenoviruses and other viruses, for that matter, is in its infancy. The emerging antiviral mechanisms of ivermectin have only recently been elucidated, paving the way for future in vivo studies on the efficacy of ivermectin in the management of viral conditions.

Anticancer activity

The versatility of ivermectin continues to unfold, with much research on its role as a potential anticancer drug. It is postulated that ivermectin can inhibit tumorigenic cell proliferation through various pathways. Researchers first noted the anticancer effects of ivermectin in 2015 through its ability to induce autophagy in cancer cells [66]. Although autophagy can be a survival mechanism for cancer cells through which damaged organelles are removed and nutrients are recycled, it has recently been shown that autophagy can also be induced by agents that suppress cancer cells [67]. Various modes of action that support the theory that ivermectin induces apoptosis in certain cancers have recently been proposed [67-70]. The potential roles of ivermectin in the management of different cancers have been explored. This includes breast cancer, gastric cancer, hepatocellular carcinoma, renal cell carcinoma, prostate cancer, leukemia, cervical cancer, ovarian cancer, glioblastoma, lung cancer, nasopharyngeal carcinoma, and melanoma [26]. The anticancer mechanism of ivermectin varies among cancers. Here, the postulated mode of action in breast cancer and glioblastoma is described. Overall, the anticancer effects of ivermectin are limited to the effects observed in human cell lines. Since this is a new horizon for ivermectin treatment, the literature evaluating this drug in human clinical trials is scarce.

Breast Cancer

Breast cancer is the leading cause of cancer among women worldwide [26]. One study revealed that after ivermectin treatment, breast cancer cell proliferation is significantly reduced in vitro and in vivo [67]. Ivermectin has been shown to inhibit the Akt/mTOR pathway, which induces autophagy in human breast cancer cell lines [26, 67]. It promotes the blockage of the Akt/mTOR pathway through ubiquitination-mediated degradation of p-21-activated kinase (PAK1) [67]. Targeting PAK1 by ivermectin may open its use in other cancers, as PAK1 is needed for growth in more than 70% of human cancers, including pancreatic, colon, prostate, and neurofibromatosis tumors in addition to breast cancer [71].

Triple-negative breast cancer, estrogen, progesterone, and human epidermal growth factor receptor 2 (HER2) negative carry the worst prognosis of breast cancer, as these are the most aggressive forms of breast cancer [26]. There is currently no known effective therapy to treat this subtype of cancer. Ivermectin has been shown to mimic the SIN-3 interaction domain (SID) to block the interaction between SID and the paired α- helix-2 [72]. Ivermectin has also been demonstrated to restore the sensitivity of triple-negative breast cancers to tamoxifen, a commonly used anticancer drug, by regulating the expression of the epithelial-mesenchymal transition (EMT)-related gene E-cadherin [72]. Given these promising findings, further investigations of the novel mechanisms of action of ivermectin in breast cancer are crucial. This could pave the way for its application as a therapeutic agent in the management of breast cancer.

Glioblastoma

Glioblastoma is one of the most lethal types of cerebral tumors, with a median survival time of 14-17 months [73]. Ivermectin has been shown to inhibit human glioblastoma cell proliferation in a dose-dependent manner [26]. Ivermectin can induce apoptosis in a caspase-dependent manner in these cells, which is related to the induction of mitochondrial dysfunction and oxidative stress [69]. Ivermectin inhibits angiogenesis by inducing apoptosis in human brain microvascular endothelial cells [69]. This allows ivermectin to prevent tumor angiogenesis and metastasis, which could be valuable anticancer effects. Ivermectin has also been demonstrated to inhibit the proliferation of these cells by blocking the Akt/mTOR pathway [69,74]. However, because ivermectin cannot cross the blood-brain barrier, its possible use is limited in the treatment of human glioblastoma.

## Conclusions

Ivermectin is now recognized as a multifaceted therapeutic with diverse potential beyond its established antiparasitic role. Although its established efficacy in combating various parasitic infections in humans and animals remains important, its therapeutic uses extend beyond. The anti-inflammatory and immunomodulatory properties of ivermectin are promising for managing inflammatory skin conditions and potentially autoimmune diseases. Its promising antiviral activity against viruses such as COVID-19 and adenoviruses, although it requires further rigorous clinical validation, presents exciting possibilities. Moreover, emerging research unveils its potential as an anticancer agent, demonstrating antiproliferative and proapoptotic effects in diverse cancer cell lines. Although these findings are encouraging in unraveling the multifunctional therapeutic potential of ivermectin, extensive in vivo studies and clinical trials are crucial to translating preclinical observations into therapeutic benefits for humans. It is important to emphasize that self-medication or the use of ivermectin outside the approved FDA indications is strongly discouraged. It is crucial to consult a healthcare professional for appropriate diagnosis and treatment.
